# Detection of Novel Actinobacillus pleuropneumoniae Serovars by Multiplex PCR: a Cautionary Tale

**DOI:** 10.1128/spectrum.04461-22

**Published:** 2023-02-01

**Authors:** Yanwen Li, Janine T. Bossé, Oliver W. Stringer, Isabel Hennig-Pauka, Preben Mortensen, Paul R. Langford

**Affiliations:** a Section of Paediatric Infectious Disease, Department of Infectious Disease, Imperial College London, London, United Kingdom; b Field Station for Epidemiology, University of Veterinary Medicine Hannover, Bakum, Germany; c Ceva Animal Health, Libourne, France; Texas A&M University

**Keywords:** *Actinobacillus pleuropneumoniae*, multiplex PCR, serotyping, serovar 9/11

## LETTER

**FIG 1:**
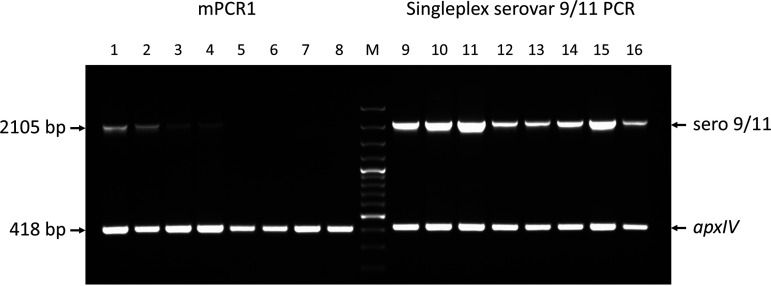
Amplicons obtained from A. pleuropneumoniae serovar 9 (CVJ13261) and 11 (56153) reference strains and clinical isolates in mPCR1 and singleplex serovar 9/11 PCR. (Left) The mPCR1 results indicated that serovar 9/11 amplicons (2,105 bp) were detected in strains CVJ13261 (lane 1) and 56153 (lane 2) and two clinical isolates (lanes 3 and 4), but not the other four other clinical isolates (lanes 5 to 8). (Right) The serovar 9/11 singleplex PCR (lanes 9 to 16, same order as for mPCR1) showed clearly visible 2,105-bp amplicons were obtained in all cases. A species-specific *apxIV* amplicon (418 bp), indicating the isolate is A. pleuropneumoniae, was detected in all cases. Lane M, molecular size markers (100-bp Plus DNA ladder; GeneRuler, Thermo Fisher Scientific).

Actinobacillus pleuropneumoniae causes porcine pleuropneumonia, a disease responsible for substantial economic losses worldwide (1). Biovar 1 and 2 isolates are NAD-dependent or NAD-independent, respectively, and there are 19 serovars based on surface carbohydrates (principally the capsule), with prevalence varying from country to country and over time (2). Serovars 1, 5, 9, 11, and 16 are considered the most virulent due to their expression of ApxI and ApxII toxins (1). Determination of serovar in a geographical location is important to identify the emergence of virulent serovars and for selection of the appropriate bacterin (whole-cell) vaccine to use (2). Due to high cross-reactivity and low reproducibility of serological-based tests, serovars are now typically determined molecularly (1–3). Based on capsule (*cps*) loci, we developed two multiplex PCRs (mPCRs) that can detect all known A. pleuropneumoniae serovars (3). mPCR1 detects the A. pleuropneumoniae species-specific *apxIV* gene and serovars 1 to 12 and 15, while mPCR2 detects *apxIV*, serovars 13, 14, and 16 to 19, and a full-length *nadV* gene that confers NAD independence and thus designation as biovar 2. Immunologically, serovar 9 and 11 isolates are highly cross-reactive, which is not surprising since the respective reference strains differ by only a single nucleotide in their serovar-specific *cpsEF* genes. Given that clinically the same management procedures would be followed (e.g., vaccine choice), we designed primers that gave the same size PCR product, with the designation serovar 9/11 (4). Isolates that were *apxIV* positive but did not have a serovar-specific band in mPCR1 were subjected to mPCR2. Since publication of the mPCRs (4), we have identified 11 clinical isolates from pigs (nine from Germany and two from The Netherlands) that were *apxIV* positive in both mPCR1 and mPCR2 but had no serovar-specific bands. This suggested that these could be new serovars. However, some of these isolates gave a barely detectable band at 2,105 bp, i.e., the size of the serovar 9/11 amplicon. Therefore, we did follow-up PCRs using the *apxIV* and serovar 9/11 primers in one tube (hereafter called 9/11 singleplex). All 11 isolates had an amplicon at 2,105 bp indicative of serovar 9/11; representative examples of conventional and aberrant isolates are shown in Fig. 1. Sanger sequencing confirmed that the amplicons were derived from serovar 9/11 biosynthetic capsule loci. The reasons for the lack of the serovar 9/11 amplicon in some isolates is not known but is most likely due to primer mismatching and/or suboptimal amplification. The serovar 9/11 amplicon is the largest in mPCR1, but increased extension times had no effect on the results (data not shown). Researchers using our mPCRs who have found amplification of *apxIV* alone in both mPCR1 and mPCR2, which can be indicative of a new serovar, are advised to additionally perform a 9/11 singleplex PCR alongside reference strains and controls. If such isolates still do not produce a 2,105-bp amplicon, then whole-genome sequence analysis should be considered to determine if the isolates represent a new serovar or disruption of a known serovar *cps* locus (e.g., by an insertion), as previously described (3). The extra PCR is likely to be of clinical utility, since serovar 9/11 and nontypeable isolates have recently been reported in Hungary (5) and Germany (6).

### Data availability.

The data supporting the conclusions drawn in the manuscript are shown in Fig. 1. All other data supporting the findings reported are available from the corresponding author on request.

## References

[1] Sassu EL, Bossé JT, Tobias TJ, Gottschalk M, Langford PR, Henning-Pauka I. 2018. Update on *Actinobacillus pleuropneumoniae*: knowledge, gaps and challenges. Transbound Emerg Dis 65:72–90. doi:10.1111/tbed.12739. 29083117

[2] Gottschalk M. 2015. The challenge of detecting herds sub-clinically infected with *Actinobacillus pleuropneumoniae*. Vet J 206:30–38. doi:10.1016/j.tvjl.2015.06.016. 26206322

[3] Stringer OW, Bossé JT, Lacouture S, Gottschalk M, Fodor L, Angen Ø, Velazquez E, Penny P, Lei L, Langford PR, Li Y. 2021. Proposal of *Actinobacillus pleuropneumoniae* serovar 19, and reformulation of previous multiplex PCRs for capsule-specific typing of all known serovars. Vet Microbiol 255:109021. doi:10.1016/j.vetmic.2021.109021. 33667982

[4] Bossé JT, Li Y, Fernandez Crespo R, Lacouture S, Gottschalk M, Sárközi R, Fodor L, Casas Amoribieta M, Angen Ø, Nedbalcova K, Holden MTG, Maskell DJ, Tucker AW, Wren BW, Rycroft AN, Langford PR. BRaDP1T Consortium. 2018. Comparative sequence analysis of the capsular polysaccharide loci of *Actinobacillus pleuropneumoniae* serovars 1–18, and development of two multiplex PCRs for comprehensive capsule typing. Vet Microbiol 220:83–89. doi:10.1016/j.vetmic.2018.05.011. 29885806 PMC6008488

[5] Sárközi R, Makrai L, Fodor L. 2018. *Actinobacillus pleuropneumoniae* serotypes in Hungary. Acta Vet Hung 66:343–349. doi:10.1556/004.2018.031. 30264610

[6] Schuwerk L, Hoeltig D, Waldmann K-H, Valentin-Weigand P, Rohde J. 2021. Sero- and *apx*-typing of German *Actinobacillus pleuropneumoniae* field isolates from 2010 to 2019 reveals a predominance of serovar 2 with regular *apx*-profile. Vet Res 52:10. doi:10.1186/s13567-020-00890-x. 33472678 PMC7818768

